# Evaluation of 1,25(OH)2D3 Effects on *FOXP3*, *ROR-γt*, *GITR*, and *CTLA-4* Gene Expression in PBMCs of Vitamin D-Deficient Women with Unexplained Recurrent Pregnancy Loss

**DOI:** 10.29252/ibj.24.5.290

**Published:** 2020-02-23

**Authors:** Elham Abdollahi, Nafiseh Saghafi, Seyed Abdolrahim Rahim Rezaee, Maryam Rastin, Lida Jarahi, Vicki Clifton, Houshang Rafatpanah

**Affiliations:** 1 Halal Research Center of IRI, FDA, Tehran, Iran;; 2 Department of Medical Immunology and Allergy, Student Research Committee, School of Medicine, Mashhad University of Medical Sciences, Mashhad, Iran;; 3 Graduate Research Trainee in Mater Research Institute-University of Queensland, Translational Research Institute, South Brisbane, Australia;; 4 Department of Gynecology Oncology, Woman Health Research Center, Mashhad University of Medical Sciences, Mashhad, Iran;; 5 Research Center for HIV/AIDS, HTLV and Viral Hepatitis, Iranian Academic Center for Education, Culture and Research (ACECR), Mashhad Branch, Mashhad, Iran;; 6 Inflammation and Inflammatory Diseases Research Center, School of Medicine, Mashhad University of Medical Sciences, Mashhad, Iran;; 7 Immunology Research Center, BuAli Research Institute, Department of Immunology and Allergy, School of Medicine, Mashhad University of Medical Sciences, Mashhad, Iran;; 8 Department of Community Medicine, School of Medicine, Mashhad University of Medical Sciences Mashhad, Iran;; 9 Pregnancy and Development, Mater Research Institute, University of Queensland, Translational Research Institute, South Brisbane, Australia

**Keywords:** FOXP3, CTLA-4, 1, 25VitD3

## Abstract

**Background:**

Vitamin D insufficiency and deficiency can be associated with adverse effects on fetus and pregnancy outcomes. This study aimed at evaluating the effect of 1,25VitD3 on specific transcription factor and markers of Tregs and Th17 cells in PBMCs of women with URPL as a case group and PBMCs of healthy women as a control group.

**Methods:**

Samples from 20 non-pregnant patients with a history of URPL were compared to 20 normal non-pregnant women. PBMCs were divided into three wells for each subject in the presence of 1,25VitD3 (50 nM, for 16 hours), PHA (10 µM; positive control), and without any treatment (negative control). By Real-time PCR (Taqman assay), specific transcription factors of Tregs and Th17 cells, *FOXP3*, *ROR-γt*, *GITR*, and *CTLA-4* mRNA expressions in two groups were measured.

**Results:**

*FOXP3/* *ROR-γt* mRNA expression in PBMCs decreased significantly in women experiencing URPL compared to the control group (*p* = 0.0001). Although 1,25VitD3 (50 nM) increased *FOXP3* gene expression (*p* = 0.0001), it did not significantly affect *ROR-γt* gene expression. Besides, 1,25VitD3 treatment significantly increased *FOXP3*/*ROR-γt* mRNA expression from baseline in PBMCs of the fetal loss group compared to that of the control group (*p* = 0.01). The 1,25VitD3 also increased *GITR* gene expression (*p* = 0.017) in PBMCs of URPL women compared to the controls.

**Conclusion:**

Vitamin D deficiency may be a contributor to recurrent pregnancy loss and suggests that the supplementation of women with Vitamin D pre-pregnancy may be protective against URPL via affecting Tregs signature genes, *FOXP3* and *GITR*.

## INTRODUCTION

**Fetus** is a **semi**-allogeneic graft; half of its major histocompatibility complex molecules comes from the **mother** and half from the father; therefore, fetus is antigenic, while the mother is immunologically responsive^[^^1 ▶^^]^. During pregnancy, the mother's immune system has to tolerate fetus, but once the immunological tolerance is broken down, recurrent pregnancy loss or spontaneous abortion may occur^[^^1 ▶^^,^^2 ▶^^]^. Recurrent fetal loss, recurrent pregnancy loss, recurrent miscarriage, habitual **abortion**, or recurrent spontaneous abortion are defined as three or more consecutive miscarriages prior to 20 weeks (or at the first trimester) of gestation^[^^3 ▶^^-^^5 ▶^^]^; approximately 1–5% of women have experienced recurrent pregnancy loss^[^^6 ▶^^]^. Although this event occurs due to several identifiable causes such as genetic, endocrine, anatomic and infectious agents, the etiology of nearly 50% of fetal loss causes is unexplained and still remains unknown; this situation is called URPL^[^^7 ▶^^-^^10 ▶^^]^.

There is a strong association between the failure of feto-maternal immunologic tolerance and recurrent pregnancy loss^[^^11 ▶^^]^. CD4^+^ T cells, which include Th1, Th2, Tregs, and Th17 cells, play an important role in the feto-maternal immune response. Maternal tolerance toward fetal alloantigens was explained by the predominant Th2-type immunity during pregnancy, while predominant Th1-type immune response was observed in recurrent pregnancy loss^[^^12 ▶^^-^^15 ▶^^]^. Th1/Th2 balance is not sufficient to define the mechanism toward tolerating the fetus. More recently, the emerging concept of the balance of Th17⁄Tregs has challenged the conventional paradigm of Th1⁄Th2 hypothesis^[16-18]^. Tregs (CD4^+^ CD25^+ ^*FOXP*3^+^) expressing CTLA-4 and GITR cells participate in the development and maintenance of tolerance in peripheral blood and tissues^[^^8 ▶^^,^^19 ▶^^,^^20 ▶^^]^. Parental antigens, human pregnancy hormones such as human chorionic gonadotropin, and chemokines are both involved in Tregs expansion^[^^21 ▶^^]^. *FOXP3* is a master regulator of Tregs that is necessary for the development and function of Tregs. Deficiency of the *FOXP3 *gene suppresses the regulatory function of Tregs^[^^11 ▶^^,^^22 ▶^^]^. Tregs contribute to the successful pregnancy via suppressing self-reactive lymphocytes. The mechanisms of actions of Tregs are mediated by a cell–cell contact (CTLA-4) and the secretion of the key cytokines such as TGF-β and IL-10^[^^23 ▶^^-^^26 ▶^^]^. 

Th17 (CD4^+^IL-17A^+^) cells are characterized by the expression of IL-17A, IL-17 F, IL-21, IL-22, IL-6, and TNF-α^[^^27 ▶^^]^. Th17 cells contribute to the host defense against both extracellular pathogens and fungal infections. *ROR-γt* is the specific transcription factor of Th17 cells^[^^28 ▶^^]^. It has been demonstrated that IL-17 is involved in the initiation or progression of inflammatory and autoimmune diseases and transplant rejection in humans^[^^29 ▶^^,^^30 ▶^^]^. Th17 cells are regulated by Tregs that play a fundamental role in the establishment and maintenance of tolerance^[^^31 ▶^^]^. Th17 cells have been reported to be likely involved in the induction of inflammation in the late but not in the early stage of abortion^[^^32 ▶^^,^^33 ▶^^]^. In the context of pathogenesis of URPL, several studies have evaluated the Th17/Tregs balance in peripheral blood, while it has been documented that local immune responses have a unique function in the fetal loss pathogenesis^[^^16 ▶^^,^^34 ▶^^-^^38 ▶^^]^.

The active form of 1,25VitD3, 1,25(OH)2D3, is a prohormone that regulates calcium hemostasis^[^^39 ▶^^]^. Conversion of vitamin D into its biologically active form, 1,25VitD3 (1,25-OH vitamin D_3_), starts in the skin and completes in the kidney by renal tubule cells^[^^40 ▶^^]^. Meanwhile, 1,25VitD3 facilitates fertilization and implantation through immunomodulatory effects at the maternal-fetal interface, promoting adequate levels of inflammatory response for decasualization and implantation, which results in successful pregnancy^[^^41 ▶^^,^^42 ▶^^]^. The deficiency of 1,25VitD3 has been associated with a higher incidence of miscarriage, preeclampsia, subfertility, infertility, and pathological alterations of critical reproductive tissues such as the endometrium^[^^43 ▶^^]^. Its deficiency is also related to gestational diabetes, bacterial vaginosis, and impaired fetal, and childhood growth and development^[^^39 ▶^^]^. It has been proposed that 1,25VitD3 influences pregnancy outcomes through its immunomodulatory impacts mediated by the VDR, a nuclear receptor, regulating gene transcription in immune cells and decidua^[^^44 ▶^^-^^46 ▶^^]^. In particular, 1,25VitD3 may regulate Th17/Tregs balance via altering the expression pattern of different genes related to these cells^[^^47 ▶^^]^.

The immune mechanisms of vitamin D effects on URPL have not yet been known completely. The placenta, ovaries, and decidua can express VDR mRNA and protein during the pregnancy^[^^46 ▶^^]^. In the context of immunomodulatory role of 1,25VitD3 in pregnancy, since a successful pregnancy is dependent on anti-inflammatory responses, it has been suggested that 1,25VitD3 could potentially be an effective treatment in URPL patients due to its immunomodulatory properties^[^^48 ▶^^,^^49 ▶^^]^. Indeed, Bubanovic^[^^48 ▶^^]^, for the first time, showed that 1,25VitD3 acts as a new potential immunotherapy agent for recurrent miscarriage patients via down-regulation of TNF-α, IL-2, and IFN-*γ* transcription. Very recently, it has been indicated that the supplements of vitamin D could increase the percentage of Tregs and *FOXP3 *gene expression while decrease the percentage of Th17 cells and *ROR-γt* gene expression *in vivo*^[^^50 ▶^^]^. The effects of 1,25VitD3 on *GITR* and *CTLA-4* genes expression has not yet been evaluated in *in* *vivo* or *in vitro* conditions. To the best of our knowledge, this is the first study evaluating the imunoregulatory effects of 1,25VitD3 on *GITR* and *CTLA-4* gene expressions in PBMCs of women with URPL. We also assessed *FOXP3 *and *ROR-γt* genes expression concurrently with *GITR* and *CTLA-4* gene expressions.

## MATERIALS AND METHODS

**Patients**

This case–control study was performed at the Mashhad University of Medical Sciences, Mashhad, Iran from 2017 to 2018. Participants included in the study were 20 women with a history of URPL as a case group and 20 fertile non-pregnant women with the history of normal deliveries (without miscarriage history) as a control group. The case and the control subjects were matched for age and BMI. For all patients and controls, at least 3 months had passed from the last fetal loss or successful pregnancy, respectively. The women in the case group did not have any other known medical conditions. Male partners underwent semen analyses; the number, shape, and movement of **sperm** were measured and were found to be normal. Both URPL and control groups were at reproductive age with regular menstruation, a normal BMI, non-pregnant as confirmed by a negative blood human chorionic gonadotropin test, and without any uterine, cervical, or genetic abnormalities.

 Women were excluded from the current study if they had a positive screening tests, including hormone tests, Treponema pallidum particle agglutination assay, human immunodeficiency virus, hepatitis B virus, hepatitis C virus, male and female karyotypes, antinuclear antibodies, anti-cardiolipin antibodies, lupus anticoagulant antibodies, antiphospholipid antibodies, fewer than three consecutive miscarriages, a positive infectious test, no deficiency in 1,25VitD3 serum level, or recent consumption of 1,25VitD3 supplement.

**Inclusion criteria**

Inclusion criteria included a negative or normal result in the routine test panel, including hormone tests, male and female karyotypes, antinuclear antibodies, antiphospholipid antibodies, anti-cardiolipin antibodies, and lupus anticoagulant antibodies. Those women who were deficient in 1,25VitD3 (less than 20 ng/ml) and had not consumed 1,25VitD3 supplement in the past three months were included in the study. 

**Isolation of PBMCs and cell culture**

Blood samples (≈10 ml) were collected from each individual at days 17-23 of the menstrual cycle and then diluted 1:1 with PBS (pH 7.4, Sigma Aldrich, USA). PBMCs were isolated from whole blood via density gradient centrifugation using the Ficoll-Hypaque (Cedarlane, Toronto, Canada). Supernatants were washed twice with PBS, re-suspended at 1 × 10^6^ PBMCs/ml in RPMI 1640 medium (BioSera, London, UK) containing 100 μ/ml of streptomycin, 100 µ/ml of penicillin, and 2 mM of glutamine and finally assessed for viability by Trypan blue exclusion. 

**Optimization of 1,25VitD3 concentration by flow cytometry assay**

To optimize vitamin D concentration, PBMCs of three patients with unexplained recurrent fetal loss were cultured in the presence of 0 (control), 10, 30, 50, and 100 nM of 1,25VitD3. Flow cytometry assessment was used for the analysis of Tregs and Th17 cells (FACS Calibur, Becton Dickinson, San Jose, CA, USA) in different time points (2, 8, 10, 12, 16, 24, and 48 hours). For cell culture experiments, treatment with 50 nM of vitamin D for 16 hours was selected as optimum for further study (data not shown). For doing the experiments, sets of 1 × 10^6^ PBMCs/patient or control were placed in each well of sterile polystyrene plates for each subject (in the case and the control groups). The experiments were conducted on uncultured PBMCS, 1,25VitD3-treated PBMCs (50 nM for 16 hours, Sigma Aldrich), and PHA (10 µM for 16 hours, Gibco, USA) or with the media only (as a negative control).

**qRT-PCR**

Total RNA was extracted from PBMCs using RNA extraction kit (Invitek, Germany) according to the instructions of the manufacturer. Reverse transcriptions were carried out by oligo(dT) primers using RevertAid™ H (Germany) primers, and probes were designed using Express software (Applied Biosystems, USA). Primer and probes specificity was checked by BLAST analysis. The sequences of the primers and probes are shown in Table 1. The quality of RNA was checked by the electrophoresis on 2% agarose gel, and the 28S, 18S, and 5.8S bands were evaluated using a UV transilluminator. The cDNA (2 µL) was amplified by real-time PCR in a final volume of 20 µL containing 10 µL of real-time PCR Master Mix (Takara, Japan), 0.3 µL of each primer, 0.3 µL of TaqMan probe, and 7.1 µL of RNase-free water using Taqman method by LightCycler 90^®^ Roch Real-Time PCR System. PCR reaction was performed as follows: 10 min at 95 ºC, followed by 45 cycles of 15 s at 95 ºC, 30 s at 57 ºC and finally 1 min at 60 ºC. Six-point 10-fold dilution standard curves were generated for *FOXP3*, *ROR-γt*, *CTLA-4*, *GITR*, using the logarithmic dilution series of the total RNA. *B2M* was included as the reference gene. The relative quantity of each mRNA was normalized to the relative quantity of β2M mRNA.

**Table 1.** Primer and probe sequences used in qRT-PCR

**Target gene**	**Sequence (5′ to 3′)**	**Product** **length (bp)**
ROR-γt	Forward: 5′-GCTAGGTGCAGAGCTTCAGG-3′ Reverse: 5′-TGTTCTCATGACTGAGCCTTGG-3′ Probe: FAM-CCTTGGCTCCCTGTCCTTCTCAGCA-BHQ	145
*FOXP3*	Forward: 5′-RACTACTTCAAGTTCCACAACATGCR-3′ Reverse: 5′-RGAGTGTCCGCTGCTTCTCTG-3′ Probe: FAM-RTCACCTACGCCACGTTCATCCGCTR-BHQ	95
β2M	Forward: 5′-TTGTCTTTCAGCAAGGACTGG-3′ Reverse: 5′-CCACTTAACTATCTTGGGCTGTG-3′ Probe: FAM-TCACATGGTTCACACGGCAGGCAT-BHQ	127
CTLA-4	Forward: 5′- CCCTGTCTTCTGCAAAGCAATGCA -3′ Reverse: 5′- CAGCCTGCCGAAGCACTGTCA -3 Probe: FAM-TCCTCGCCCGAAGAGCGCGG-BHQ	114
GITR	Forward: 5′-GACGAGTTTGTGGACTCCTTTAAG-3′ Reverse: 5′-CCTGCCCAAGTTCCGATCC-3′ Probe: FAM-TCCTCGCCCGAAGAGCGCGG-BHQ	129

**Statistical analysis**

All statistical analyses were carried out using the SPSS 16.0 software and Graphpad Prism 5.0. Data were presented as mean ± SE. For comparisons of the mean data, parametric *t*-test and ANOVA were performed. *p* value of less than 0.05 was considered statistically significant.

**Ethical statement**

The above-mentioned sampling protocols were approved by the Research Ethics Committee of Mashhad University of Medical Sciences, Mashhad, Iran (ethical code: 961711). Written informed consents were provided by all the patients.

## RESULTS

Clinical characteristics of the patients with recurrent fetal loss are shown in Table 2. Based on the Table, there were no significant differences in age and BMI between the two groups. The mean age and BMI of the patients with unexplained recurrent fetal loss were 23.3 ± 6.00 and 24.3 ± 5.00 years, and those of the control group were 25.6 ± 6.00 (*p* = 0.71) and 23.5 ± 6.03 (*p* = 0.51), respectively. Sex hormones levels in the studied groups are represented in Table 3. There were no significant differences in the serum level of progesterone, estradiol, prolactin, FSH, or LH between the case and control groups. 

**Table 2.** Clinical characteristics of women with URPL

**Clinical characteristics**	**Control ** **(n = 20)**	**Patients with unexplained recurrent fetal loss (n = 20)**
Age (y)	23.3 ± 6.00	24.3 ± 5.0
BMI	24.3 ± 6.00	23.5 ± 6.03
Mean of miscarriage	4 (rang = 3-6 miscarriage)	-
Mean of time of miscarriage (week of gestation)	6.4	-
Mean of time of last miscarriage (months passed from last miscarriage)	9.2	-
Blood group (%)		
A	15.8	33.3
B	47.4	22.2
AB	10.5	11.1
O	26.3	33.3

**Table 3:** Sex hormones levels in the case and control group

**Hormone**	**Case group** **(n = 20)**	**Control group** **(n = 20)**	***p*** **value**
E2 (pg/ml)	217.8 ± 141.5	221.8 ± 156	0.464
Progesterone (ng/ml)	5.3 ± 6.8	5.9 ± 6.2	0.579
Prolactin (ng/ml)	20.7 ± 22.8	19.5 ± 20.6	0.365
FSH (mIU/ml)	5.9 ± 3.7	4.5 ± 2.8	0.573
LH (mIU/ml)	14.9 ± 16.2	14.1 ± 18.3	0.854

**Fig. 1:**
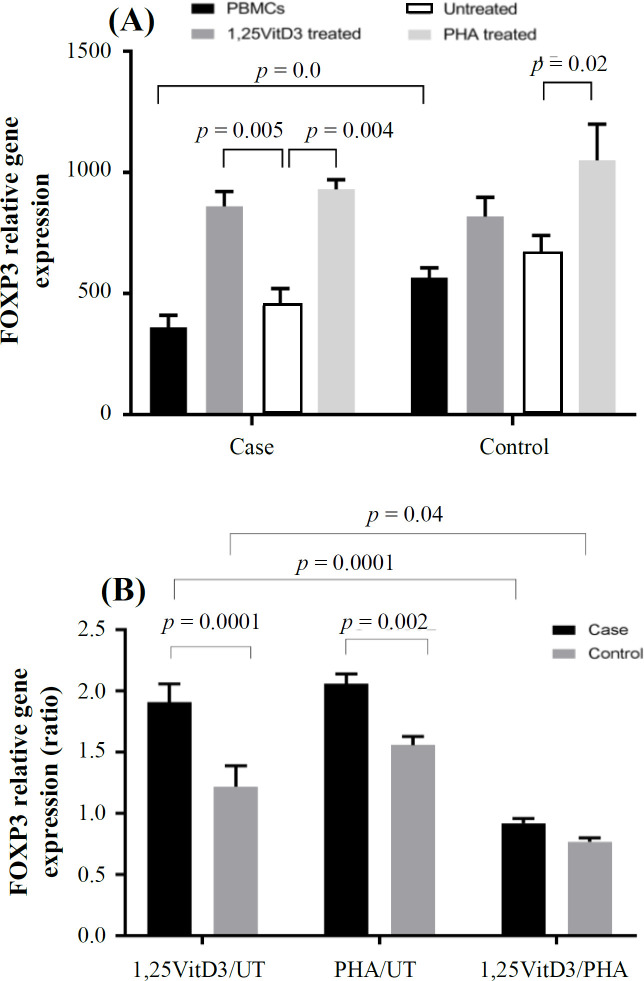
*FOXP3* gene expression. *FOXP3* gene expression (A) and its ratio (B) in the presence of 1,25VitD3, PHA (as a positive control), and negative control (as a baseline) in PBMCs of patients with URPL and the controls. UT, untreated

**Expression of Tregs and Th17 transcription factors in PBMCs**

The expressions of *FOXP3 *and* ROR*-γt genes were measured in PBMCs of the unexplained recurrent fetal loss group and control group by qRT-PCR.

**Fig. 2:**
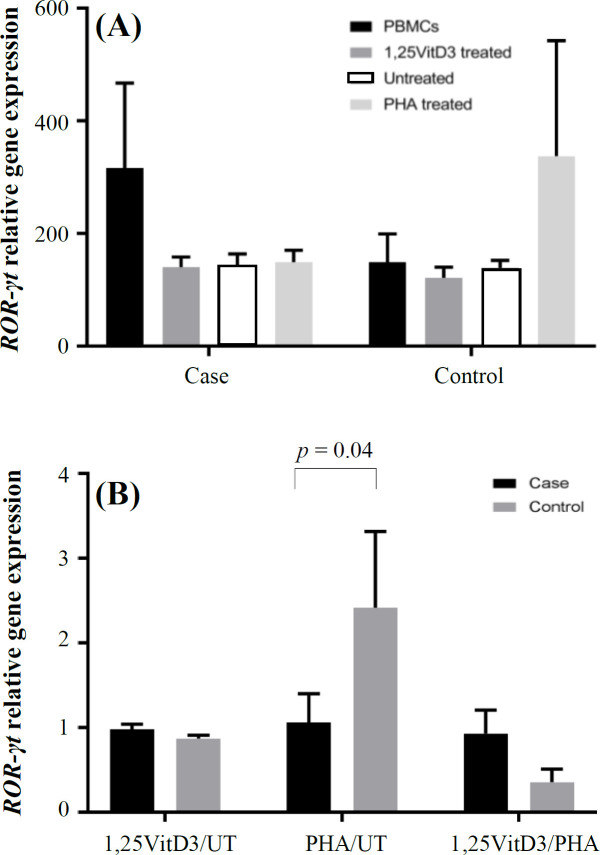
ROR-γt gene expression. ROR-γt gene expression (A) and its ratio (B) in the presence of 1,25VitD3, PHA (as  a positive control), and negative control (as a baseline) in PBMCs of patients with URPL and the controls. UT, untreated

**Fig. 3:**
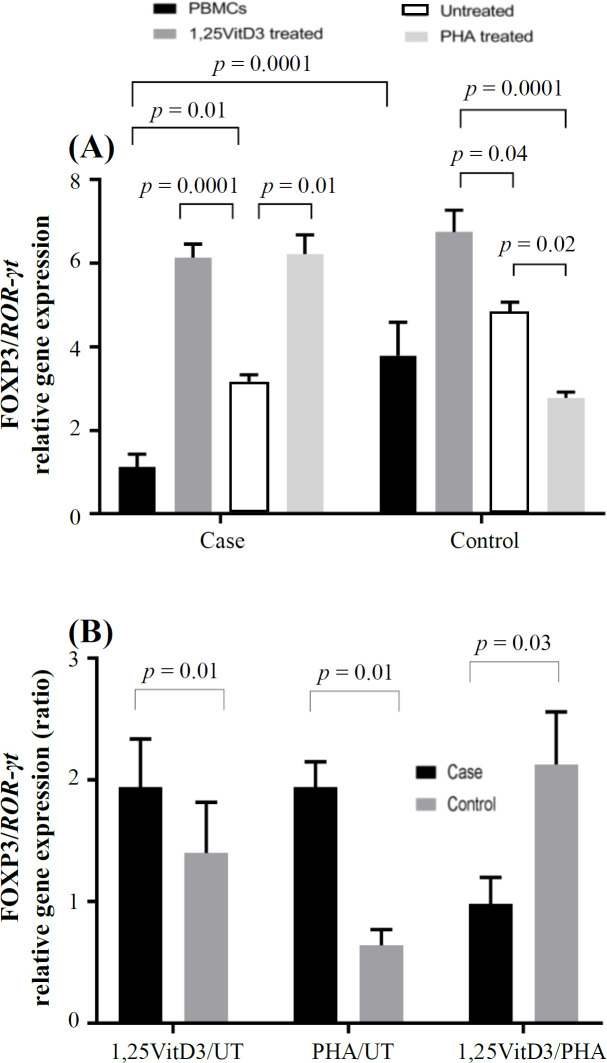
*FOXP3/ROR*-γt gene expression. *FOXP3/ROR*-γt gene expression (A) and its ratio (B) in the presence of 1,25VitD3, PHA (as a positive control), and negative control (as a baseline) in PBMCs of patients with URPL and the controls. UT, untreated

Expressions of these transcription factors were then divided by reference gene (B2M) to normalize the results and reach a normalized index for each gene of interest for each sample. *FOXP3* gene expression decreased significantly in patients with fetal loss compared to the control group (360.60 ± 50.50 vs. 565.95 ± 40.36; *p* = 0.02; Fig. 1A). Gene expression index of *ROR-γt* in PBMCs of URPL women was non-significantly higher than the control group (316.41 ± 150.99 vs. 148.94 ± 50.21; *p* > 0.05; Fig. 2B). Our results also showed that *FOXP3/ROR-γt* ratio was significantly lower in PBMCs of the case group than that of the control group (1.13 ± 0.3 vs. 3.79 ± 0.8; *p* = 0.0001; Fig. 3A).

**Expression of Tregs markers in PBMCs**

**Fig. 4:**
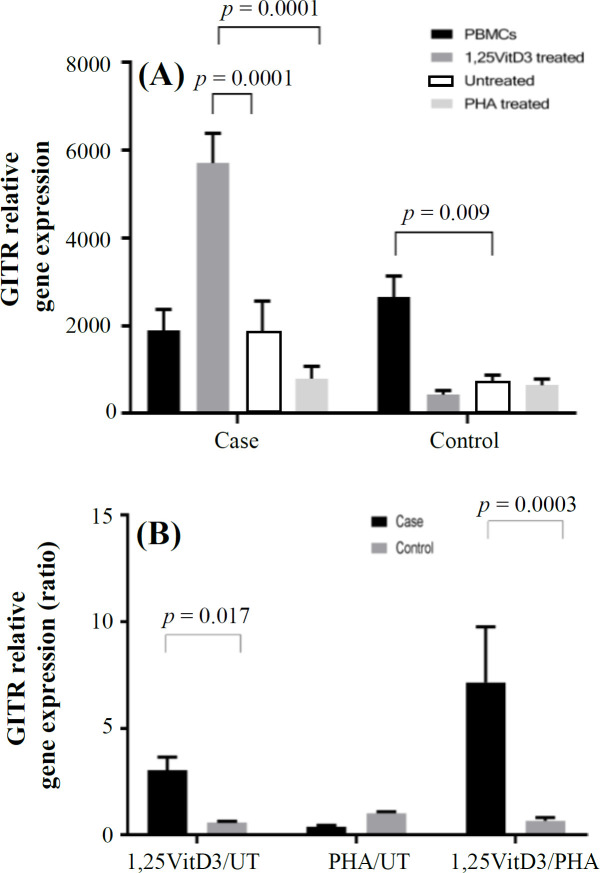
GITR gene expression. GITR gene expression (A) and its ratio (B) in the presence of 1,25VitD3, PHA (as a positive control), and negative control (as a baseline) in PBMCs of patients with URPL and the controls. UT, untreated

**Fig. 5:**
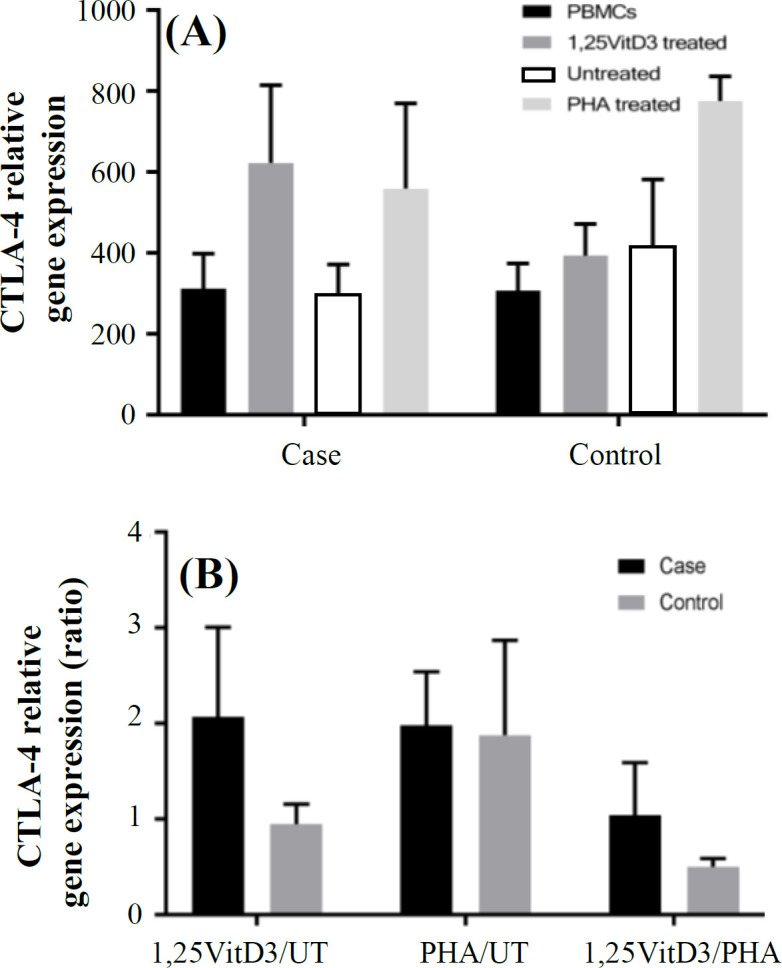
CTLA-4 gene expression. CTLA-4 gene expression (A) and its ratio (B) in the presence of 1,25VitD3, PHA (as a positive control), and negative control (as a baseline) in PBMCs of patients with URPL and the controls. UT, untreated

*GITR* gene expression in PBMCs of patients with URPL decreased compared to the controls, but this change was not significant (1891.60 ± 479.14 vs. 2647.3 ± 483.39; *p* > 0.05; Fig. 4A). There was also no significant difference in *CTLA-4* gene expression in PBMCs treatment with 1,25VitD3 between cells from women with fetal loss and the control group (311.21 ± 78.65 vs. 307.11 ± 67.39; *p* > 0.05; Fig. 5A). 

**Effects of 1,25VitD3 on the expression of Tregs and Th17 transcription factors in PBMCs**

Our results showed that PBMCs treatment with 1,25VitD3 increased *FOXP3* gene expression from the baseline (untreated PBMCs) in women with URPL compared to the controls (1.91 ± 0.15 vs. 1.22 ± 0.1; *p* = 0.0001; Fig. 1B). The greater effect of 1,25VitD3 on *FOXP3* gene expression in PBMCs of women with URPL may be related to the impairment of Tregs function in women with URPL, which could be strengthen by the treatment of PBMCs with 1,25VitD3 in such women. In the case group, there was a significant increase in *FOXP3* gene expression in the PBMCs in the presence of 1,25VitD3 in comparison with the untreated PBMCs (860.84 ± 60.89 vs. 450.02 ± 70.47; *p* = 0.005; Fig. 1A). In the control group, no significant change was observed in *FOXP3 *gene expression in the PBMCs in the presence of 1,25VitD3 in comparison with the untreated PBMCs (817.97 ± 80.51 vs. 670.47 ± 70.55; *p* > 0.05; Fig. 1A). There was also no significant difference in *ROR-γt* gene expression in treatment with 1,25VitD3 between cells from women with fetal loss and the control group (0.98 ± 0.06 vs. 0.87 ± 0.04; *p* > 0.05; Fig. 2B). In the case and control groups, no significant changes was found in *ROR-γt* gene expression in the PBMCs in the presence of 1,25VitD3 in comparison with the untreated PBMCs (140.09 ± 18.00 vs. 142.24 ± 21.39 and 121.25 ± 19.00 vs. 138.99 ± 13.47, respectively; *p* > 0.05; Fig. 2A). The 1,25VitD3 significantly affected *FOXP3/ROR-γt* ratio in PBMCs women with URPL compared to that of the controls (1.94 ± 0.50 vs. 1.40 ± 0.42; *p* = 0.01; Fig. 3B). PHA was used as a positive control and demonstrated that the cells are responsive to a stimulus, and that response does not vary between the groups.

**Effects of 1,25VitD3 on the expression of Tregs markers in PBMCs**

The results indicated that *GITR* gene expression increased in the presence of 1,25VitD3 from baseline (untreated PBMCs) in the peripheral blood in patients with fetal loss (3.05 ± 0.51 vs. 0.59 ± 0.10; *p* = 0.017; Fig. 4B). In the case group, there was a significant increase in *GITR* gene expression in the PBMCs in the presence of 1,25VitD3 as compared with the untreated PBMCs (5700.47 ± 681.89 vs. 1863.02 ± 700.47; *p* = 0.005; Fig. 4A). In the control group, no significant shift was found in *FOXP3 *gene expression in PBMCs in the presence of 1,25VitD3 in comparison with the untreated PBMCs (433.30 ± 90.10 vs. 723.63 ± 150.03; *p* > 0.05; Fig. 4A). The 1,25VitD3 non-significantly increased the gene expression of *CTLA-4* in the fetal loss group compared to the control group (2.07 ± 0.94 vs. 0.95 ± 0.21; *p* > 0.05; Fig. 5B). PHA, as a positive control, demonstrated that the cells are responsive to a stimulus, and that response does not vary between the groups. In the case and control groups, there were no significant changes in *CTLA-4* gene expression in the PBMCs in the presence of 1,25VitD3 in comparison with the untreated PBMCs (623.27 ± 191.80 vs. 299.74 ± 72.50 and 393.85 ± 78.04 vs. 412.28 ± 169.68, respectively; *p* > 0.05; Fig. 5A).

**Correlation between** ***FOXP3*** **and** ***CTLA-4*** **gene expression**

**Fig. 6:**
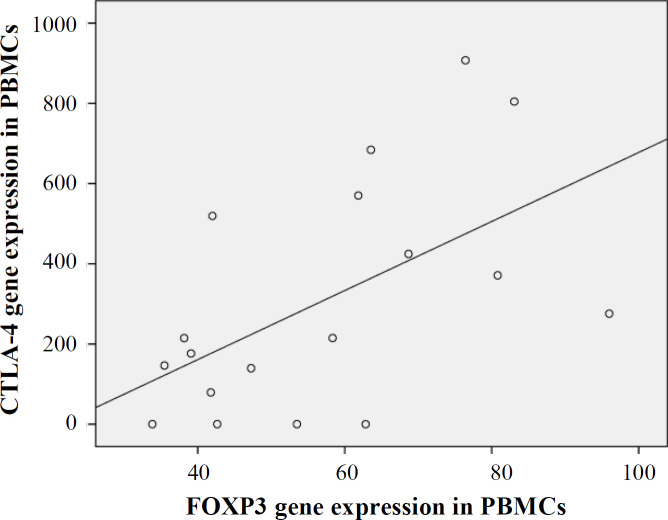
Direct significant correlation between FOXP3 and CTLA-4 gene expression in the control group

There was a direct significant correlation between *FOXP3* and *CTLA-4* in the control group (*p=* 0.01, R= 0.55, Fig. 6).

## DISCUSSION

Fetus is an allograft to the maternal host during early pregnancy, and failure in immune tolerance can lead to fetal loss^[^^51 ▶^^,^^52 ▶^^]^. Alteration of Tregs/Th17 balance in patients with fetal loss causes a rise in inflammatory responses that play a probable role in the pathogenesis of fetal loss^[^^53 ▶^^-^^55 ▶^^]^.

Tregs (CD4^+^ CD25^+^high FOXP3^+^) expressing *CTLA-4* and *GITR* are necessary to mediate maternal tolerance to the fetus^[^^56 ▶^^]^. Previous studies have shown an increasing number of Tregs in peripheral blood, and decidua was due to the recognition of the semi allograft fetus antigens by Tregs, a process resulting in Tregs expansion^[^^41 ▶^^,^^57 ▶^^-^^59 ▶^^]^. In both mice and humans, it has been supposed that the highest percentage of Tregs would reach the second trimester of pregnancy and then diminish in the postpartum period^[^^38 ▶^^,^^57 ▶^^,^^60 ▶^^,^^61 ▶^^]^. Defects in the numbers or functions of Tregs have been observed in women with recurrent pregnancy loss and mouse models in peripheral blood as well as in deciduas^[^^8 ▶^^,^^37 ▶^^,^^40 ▶^^,^^59 ▶^^,^^61 ▶^^,^^62 ▶^^]^. Human TH17 cells producing IL-17 may have a major role in rejecting fetal antigens and, therefore, may be harmful to the maintenance of pregnancy^[^^35 ▶^^,^^63 ▶^^,^^64 ▶^^]^. *ROR-γt*, as a specific transcription factor of Th17 cells, is required for the initiation and stabilization of the Th17 phenotype^[^^28 ▶^^]^.

In the context of immunomodulatory role of 1,25VitD3 in pregnancy, since a successful pregnancy is dependent on anti-inflammatory responses, it has been suggested that 1,25VitD3 could potentially be an effective treatment in URPL patients due to its immunomodulatory properties^[^^48 ▶^^,^^49 ▶^^]^.

For our study, we analyzed the expression of *FOXP3*, as a master regulator of Tregs, two surface markers of these cells (*GITR* and *CTLA-4*), and *ROR-γt*, as Th17 cells transcription factor, in PBMCs of URPL women and women with the history of at least successful pregnancy as a control group.

We showed that *FOXP3* gene expression decreased significantly in peripheral blood in woman with URPL, while there was no significant change in *ROR-γt *gene expression compared to the controls. *FOXP3/ROR-γt* ratio was significantly lower than that of PBMCs of women experiencing URPL compared to the control group (Figs. 1A, 2A, and 3A). This finding is in agreement with other studies, indicating that *FOXP3 * gene expression decreased in patients with pregnancy loss^[^^60 ▶^^,^^65 ▶^^]^. The results of the present study indicated that 1,25VitD3 treatment could significantly increase *FOXP3 * gene expression (Fig. 1B) in PBMCs of women with URPL. Besides, the reduced percentage of Tregs in PBMCs of women with URPL was observed and elevated upon treatment with 1,25VitD3 (unpublisheddata). The results of the present study indicated that 1,25VitD3 treatment could significantly increase *FOXP3* gene expression (Fig. 1B) in PBMCs of women with URPL. Herein, we showed a reduction in the percentage of Tregs in PBMCs of women with URPL, and 1,25VitD3 treatment elevated the percentage of Tregs (unpublished date). It is probable that the reduction of Tregs in PBMCs of URPL women is closely related to the decreased expression of *FOXP3*^[^^35 ▶^^]^. It is probable that the reduction of Tregs in URPL women is closely related to the decreased expression of *FOXP3*^[^^41 ▶^^]^. Therefore, we speculate that 1,25VitD3 may increase the percentage of Tregs and *FOXP3* gene expression in pregnancy as shown in other study^[^^66 ▶^^]^.

There are some contradictory results about the effects of 1,25VitD3 on Tregs numbers^[^^67 ▶^^-^^69 ▶^^]^. 1,25-(OH)2D3 may promote Tregs differentiation and enhance the frequency of activation-induced *FOXP3*^+^ T cells, and this was dependent on the presence of IL-2 in culture. These *FOXP3*^+^ Tregs expressed high levels of CTLA-4, an inhibitory receptor^[^^67 ▶^^]^. However, a comparable study using naïve murine T cells has suggested that 1,25-(OH)2D3 inhibits both IL-17 and Tregs differentiation *in vitro*. Mayne *et al.*^[^^68 ▶^^]^ have indicated that 1,25VitD3 could confer protection against an EAE model in mice through decreasing Th1 and Th17 (with abundant VDR transcripts). This study reflects that low VDR expression in CD4^+^FOXP3^+^ Tregs may allow them to escape sensitization to apoptotic signals, leading to unchnaged Tregs proportion in the presence of 1,25VitD3 in an EAE model. Some early studies have suggested that vitamin D has deleterious effects on allergic airway disease. VDR-deficient mice failed to develop experimental allergic asthma, leading the authors to suggest a role for vitamin D in driving Th2 inflammation in the airways^[^^69 ▶^^]^. Nevertheless, considerable interest remains in the therapeutic application in asthma, and examples of beneficial effects exist. The probable reason behind the lack of any significant difference between the *ROR*-γt gene expression in the case and control groups is that the regulation of the immune response by vitamin D is achieved through increasing *FOXP3* gene expression and enhancing Tregs function, which results in the inhibition of inflammatory responses of Th17 cells, contributing indirectly in recurrent spontaneous abortion. In addition, according to our unpublished flowcytometry data, 1,25VitD3 up-regulated the differentiation of Tregs *in vitro* while did notaffect the Th17 cell differentiation. Therefore, we suppose that the lack of any significant effect on Th17 percentage comes from no impaction on *ROR*-γt gene expression by 1,25VitD3, suggesting that 1,25VitD3 is an immunemodulatory agent regulating Tregs differentiation to ensure pregnancy progresses to term. Vitamin D could act to prevent recurrent abortion as a regulator agent of immune responses rather than an immunosuppressive of the inflammatory responses.

Our findings, for the first time, demonstrated that 1,25VitD3 enhanced *GITR* gene expression in PBMCs of women with URPL (Fig. 4B). *GITR* is a marker characteristic for Tregs. High level of *GITR* expression can be obtained in 72. Along with the regulation of Tregs reactivity, *GITR* induces co-stimulatory signals in Tregs involved in T cell proliferation and cytokine production^[^^72 ▶^^]^. Our finding also indicated that there was a direct significant correlation between *FOXP3* and *CTLA-4* gene expression in the control group. Therefore, it can be concluded that Tregs FOXP3^+^CTLA^+^ function may disrupt in patients with URPL. According to our results, 1,25VitD3 had no significant effect on *CTLA-4* gene expression. Meanwhile, the evaluation of the effects of 1,25VitD3 on Tregs and Th17 cells and related signaling pathways involved in differentiation of Tregs and Th17 cells should be considered in future studies to understand more details of these.

To sum up, the present study revealed that 1,25VitD3 may directly affect the immunoregulatory mechanism through increasing *FOXP3* and *GITR* gene expression rather than immunosuppression effect on Th17 cells. The present study was not without limitations, and we propose that future studies are needed to be performed with the larger sample size. Considering the role of Th17 and Tregs in pregnancy outcome, targeting this ratio may result in a successful pregnancy. Therefore, 1,25VitD3 may have a function in providing requirements for the anti-inflammatory state and promoting pregnancy maintenance. Determination of the optimal time and dosage of 1,25VitD3 are very important for a desirable clinical outcome. 
